# A Derivative-Based Framework for Real-Time Signal Processing and Event Detection in Impedance Flow Cytometry

**DOI:** 10.3390/s25237252

**Published:** 2025-11-27

**Authors:** Brendan Wurts, Charlie Jindrich, Yu Gong, Joshua Ojih, Ming Hu, Kun Yin, Leilei Shi

**Affiliations:** 1 Department of Engineering, School of Engineering, Computing, and Mathematics, College of Charleston, Charleston, SC 29424, USA; wurtsbs@g.cofc.edu (B.W.); jindrichca@g.cofc.edu (C.J.); 2 Department of Physics and Astronomy, School of Natural and Environmental Sciences, College of Charleston, Charleston, SC 29424, USA; gongy@cofc.edu; 3 Department of Mechanical Engineering, University of South Carolina, Columbia, SC 29208, USA; jojih@email.sc.edu (J.O.); hu@sc.edu (M.H.); 4 School of Global Health, Chinese Center for Tropical Diseases Research, Shanghai Jiao Tong University School of Medicine, Shanghai 200025, China; kunyin@sjtu.edu.cn

**Keywords:** impedance flow cytometry, derivative, signal processing, event detection

## Abstract

Impedance flow cytometry (IFC) enables label-free, real-time characterization of cells and particles, but its performance depends critically on accurate event detection and feature extraction under varying noise and acquisition conditions. Conventional pipelines typically rely on multi-stage thresholding, wavelet transforms, template-based correlation methods, or neural-network models. These approaches generally require additional preprocessing steps and involve multiple parameters or hyperparameter tuning. In this work, we present a simple derivative-based signal processing framework that enables baseline-drift suppression, event detection, and feature extraction within a single computational step. The derivative approach improved precision and recall by approximately 20% and reduced the false discovery rate by 15–25% compared with simple thresholding, while requiring only 22–55% of the processing time across all the test conditions. The algorithm operates in linear time with minimal memory overhead and does not rely on template matching or trained parameters, making it well-suited for real-time or embedded, resource-constrained IFC platforms. We further demonstrate that derivative-extracted features enable accurate real-time classification of microparticles, achieving >98% accuracy while maintaining a processing speed that is approximately two orders of magnitude faster than the data-acquisition rate.

## 1. Introduction

The analysis of single-cell flow cytometry data plays a pivotal role in biomedical research, providing critical insights into cellular characteristics and functions. Flow cytometry, and in particular impedance flow cytometry, is a versatile technique that measures the electrical impedance of cells or particles as they transit through a microfluidic channel [1]. Unlike traditional optical flow cytometry, which relies on fluorescence markers, IFC offers a label-free, real-time approach to analyze cell properties [2]. This technology is essential for a broad range of biomedical applications, including cell counting, sizing, and phenotyping, which are crucial in fields such as oncology, hematology, and immunology [3,4,5,6,7,8,9]. Moreover, IFC’s ability to rapidly process large volumes of cells with minimal sample preparation makes it a valuable tool for high-throughput diagnostic and therapeutic applications.

**Figure 1:**
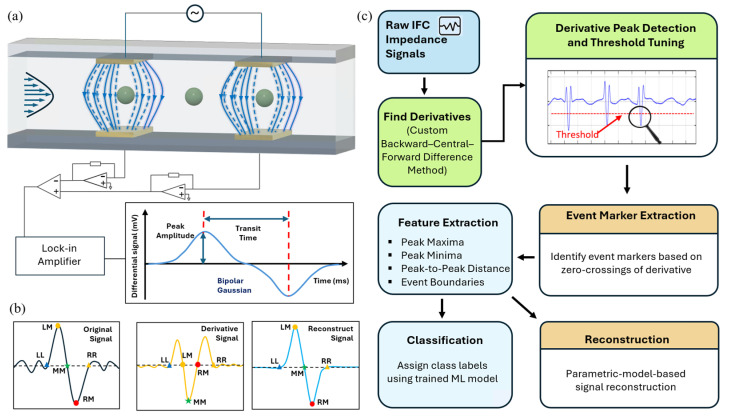
System architecture and derivative-based IFC processing workflow. (**a**) Schematic of the microfluidic impedance detection system; (**b**) Illustration of the derivative-based method: raw signal, first derivative with peak locations, and reconstructed bipolar Gaussian signal using extracted features; (**c**) End-to-end data processing pipeline including derivative computation, event detection, feature extraction, classification, and reconstruction.

In typical IFC setups (as shown in Figure 1a), a microfluidic channel equipped with two pairs of electrodes detects impedance changes as cells or particles pass through. As particles traverse the detection region, the system generates a characteristic double-peaked signal with one positive peak and one negative peak. This bipolar waveform reflects the variation in impedance as the particles enter and exit the electrode field, respectively [10,11,12]. The most commonly used technique for acquiring impedance signals in microfluidic cytometry relies on lock-in amplifiers, which offer high sensitivity by demodulating a sinusoidal carrier signal at a fixed frequency [13,14,15,16]. In these systems, the target impedance is excited with a known AC voltage, and the resulting current is converted to a voltage using a transimpedance amplifier. The lock-in amplifier multiplies this voltage with a synchronized reference signal to shift the frequency content to DC, followed by a low-pass filter that extracts the impedance envelope while suppressing higher harmonics and out-of-band noise. This hardware-level filtering effectively attenuates high-frequency noise while preserving low-frequency impedance signals.

Following the acquisition, two key steps are typically performed: event detection and feature extraction. Those stages are often performed with proprietary or custom-built algorithms, yet systematic performance evaluations are seldom provided. Most commonly, simple threshold-based peak-finding approaches are employed for the event-detection stage [17,18,19,20]. While conceptually straightforward and computationally efficient, threshold-based event detection suffers from several critical limitations: it is highly sensitive to baseline drift, requires careful parameter tuning, and is vulnerable to noise. As a result, a preliminary denoising step is often necessary. For this reason, some alternative strategies have been introduced to improve robustness. Wavelet-based detection [21] leverages the time–frequency decomposition of the signal to isolate transient features resembling bipolar impedance peaks, offering improved noise tolerance but relying heavily on the choice of wavelet function. Correlation-based methods [10] detect events by correlating the signal with a predefined reference waveform, providing greater sensitivity to subtle waveform variations but introducing dependence on the fidelity of the template, which may vary with particle type, flow conditions, or electrode geometry. Odd-symmetric correlation methods [11] simplify this approach by exploiting the intrinsic symmetry of differential signals, reducing template mismatch risks while retaining sensitivity to waveform morphology. More recently, neural network-based approaches have been explored for event detection and signal denoising in impedance cytometry, demonstrating strong performance in noisy or complex conditions [22,23,24]. However, these methods typically require large and annotated datasets, involve higher computational costs, and often operate as ‘black boxes’, limiting their interpretability and integration into embedded, real-time cytometry platforms. After event detection, the next critical step is feature extraction, where signal characteristics such as peak amplitude and transit time are quantified for downstream classification. In many pipelines, this requires an additional layer of processing—such as derivative-based peak localization [17] or template matching [25]—to identify the local maxima and minima within the detected event window. While effective, these added operations increase the overall processing complexity and can impact real-time performance, especially in resource-constrained implementations.

Derivative-based filters have a long history in biomedical signal processing. Classical ECG QRS detectors and many modern PPG peak-detection algorithms rely on derivative or slope-based transforms followed by simple thresholding to achieve robust event detection under noisy and low-SNR conditions [26,27,28]. Although several groups have employed derivative or slope information in microfluidic impedance cytometry, these operations are typically employed only as a local aid—for example, to refine extrema detection [17] or help resolve closely spaced resistive-pulse events [29]. As noted by Honrado et al. [5], derivative-based operations are part of the standard toolbox for detecting local maxima in impedance-cytometry signals.

In this paper, we extend the role of derivatives beyond local feature extraction and present a derivative-based framework that integrates baseline-drift suppression, event detection, and feature extraction within a single algorithm. In our approach, the derivative itself serves as the primary detection signal, enabling simultaneous drift suppression, boundary identification, and feature extraction within a single step. An optional lightweight recovery function is further included to facilitate rapid visual verification of detected events, improving interpretability and user confidence. The integrated structure of the algorithm improves both efficiency and accuracy compared with simple threshold-based approaches, which remain common in IFC signal processing but typically require multiple independent processing stages [17,18,19,20]. In a typical setting, our approach requires only about 22–55% of the processing time compared with multi-stage thresholding pipelines. At the same time, precision and recall improve by approximately 20%, and the false discovery rate decreases by 15–25%. Compared with more advanced wavelet-, correlation-, or neural-network-based techniques, the proposed approach preserves the key advantages of simplicity and speed: it operates in linear time with minimal memory overhead, requires no multi-scale transforms or template matching, and does not rely on trained parameters.

Building on this computational efficiency, we further examine the compatibility of this framework with machine learning workflows, showing that the derivative-based features serve as effective inputs for downstream classification models. Using synthetic datasets of 4 µm and 7 µm beads, the system achieved accuracy and recall exceeding 98.6% for both bead sizes under a single-frequency measurement, with a total processing time that is two orders of magnitude faster than its acquisition rate. For experimental validation with the same bead sizes, the extracted features formed tightly clustered and well-separated groups, closely matching the synthetic results. These findings demonstrate both the robustness of the proposed approach under realistic measurement conditions and its suitability for real-time execution.

## 2. Materials and Methods

### 2.1. Workflow Description

As illustrated in Figure 1c, the workflow consists of five core modules: (1) derivative computation using a finite-difference scheme; (2) threshold-based peak detection to identify high-slope signal transitions; (3) event marker extraction via derivative zero-crossings; (4) feature extraction and classification; and (5) signal reconstruction using parametric signal models, such as a bi-Gaussian model for spherical particles. Each stage of the workflow is modular, enabling easier customization and future enhancements to individual components without affecting the entire pipeline.

The core of the proposed derivative-based method involves computing the first derivative of the raw impedance signal to capture rapid transitions associated with the passage of individual cells or particles through the detection region. To ensure accurate and stable derivative computation across the entire signal, the algorithm utilizes finite-difference schemes—applying forward and backward differences at the boundaries and central differences for interior points. Following derivative computation, a thresholding step is applied to suppress background noise while preserving high-slope transitions indicative of true events. As illustrated in Figure 1b, key signal landmarks such as zero crossings and derivative extrema are then extracted. These features enable segmentation of the signal into meaningful events, with each event flanked by left and right zero-crossings. Within each segmented event window, biophysical features such as peak-to-peak amplitude and transit time are extracted using the detected landmarks and the corresponding values from the original signal. These features were then supplied to a lightweight decision tree classifier—trained on synthetically labeled IFC data—to assign each detected event to predefined particle classes.

The optional reconstruction procedure employs a parametric signal model that approximates each event waveform according to its characteristic morphology. For spherical particles, a bi-Gaussian model is applied, representing the event as two opposing Gaussian components—one positive and one negative—symmetrically positioned around the event center and parameterized by amplitude and width [11,30]. Notably, for events involving non-spherical or irregular particles, alternative or modified parametric models may be required to accurately capture the signal morphology. The final reconstructed signal is generated by aggregating contributions from all detected events, yielding a smoothed approximation of the original waveform.

### 2.2. Data Generation and Collection

To comprehensively evaluate the performance and robustness of the derivative-based signal processing method, we utilized both synthetic and experimental IFC datasets. To evaluate the performance of the derivative method under real experimental conditions, we used three types of polystyrene beads with diameters of 2.07 μm (catlog number FSDG005), 4.12 μm (catalog number FSDG006) and 7.32 μm (catalog number FSDG007), both sourced from Bangs Laboratories, Inc. (Fishers, IN, USA). If not otherwise specified, the polystyrene bead suspension was diluted to a concentration of 1 × 10^6^ beads per milliliter in a 1 × PBS buffer solution, obtained from Thermo Fisher Scientific (Waltham, MA, USA). All the experiments were conducted using a microfluidic chip (Micronit, Enschede, The Netherlands) with a 30 μm × 30 μm channel containing two pairs of facing platinum (Pt) electrodes (20 μm width, 20 μm spacing). The particle suspension flowed through the channel at 4 μL/min using a flow rate controller from Fluigent (Fontainebleau, France).

Signal detection was performed with an HF2LI lock-in amplifier and an HF2TA trans-impedance amplifier module, both from Zurich Instruments (Technopark, Zurich, Switzerland). A 1 MHz alternating current (AC) excitation signal with a peak amplitude of 1 V was applied across the electrode pairs to induce impedance changes during particle transit. The resulting signal was demodulated using a fourth-order low-pass filter with a 500 Hz bandwidth to isolate the magnitude envelope. Data were digitized at a sampling rate of 115.1 kSa/s, saved in MATLAB (.mat) format, and subsequently post-processed using custom MATLAB scripts (MATLAB R2023b).

Synthetic data streams were generated based on the previous report [30], and a custom MATLAB script was designed to emulate realistic IFC signals under our experimental conditions. Each 30 s data stream consisted of a sequence of synthetic events modeled as bipolar Gaussian pulses, capturing the characteristic transients generated by individual cell or particle passages. Event arrivals were randomized according to a Poisson distribution, consistent with the stochastic nature of particle flow in microchannels [11,31]. Additive white noise with standard deviation σ_N_ was incorporated into the data stream, followed by an n-stage first-order filter producing a final bandwidth of 500 Hz. A sampling frequency of 115.1 kSa/s was applied. Table 1 summarizes the parameter values used to generate synthetic datasets, which reflect typical experimental settings. Unless otherwise noted, these values were used as default settings for all simulations. Particle diameters from 2 to 7 μm were modeled with a 5% coefficient of variation (CV) around the nominal size and a concentration of 1 × 10^3^ μL^−1^. A flow rate of 2 μL/min was used. Frequency-domain and time-domain analysis confirmed that the spectral characteristics of the synthetic signals closely matched those observed in experimental recordings.

### 2.3. Real-Time Processing and Classification

To evaluate the real-time applicability of the derivative-based signal processing method, we implemented a complete processing pipeline that analyzes streaming IFC signals from raw acquisition through classification in MATLAB R2023b.

For each data stream, complex demodulated impedance signals were generated and converted to magnitude form, which served as the input to the derivative-based detection algorithm. The outputs were visualized by overlaying them on the raw signal trace in real time, with color-coded markers indicating their predicted class membership. All predicted events were subsequently matched to their ground-truth counterparts using a fixed temporal tolerance, enabling the computation of precision, recall, F1-score, and other performance metrics across multiple data streams.

## 3. Results and Discussion

### 3.1. Derivative-Based Event Detection and Signal Reconstruction

**Figure 2:**
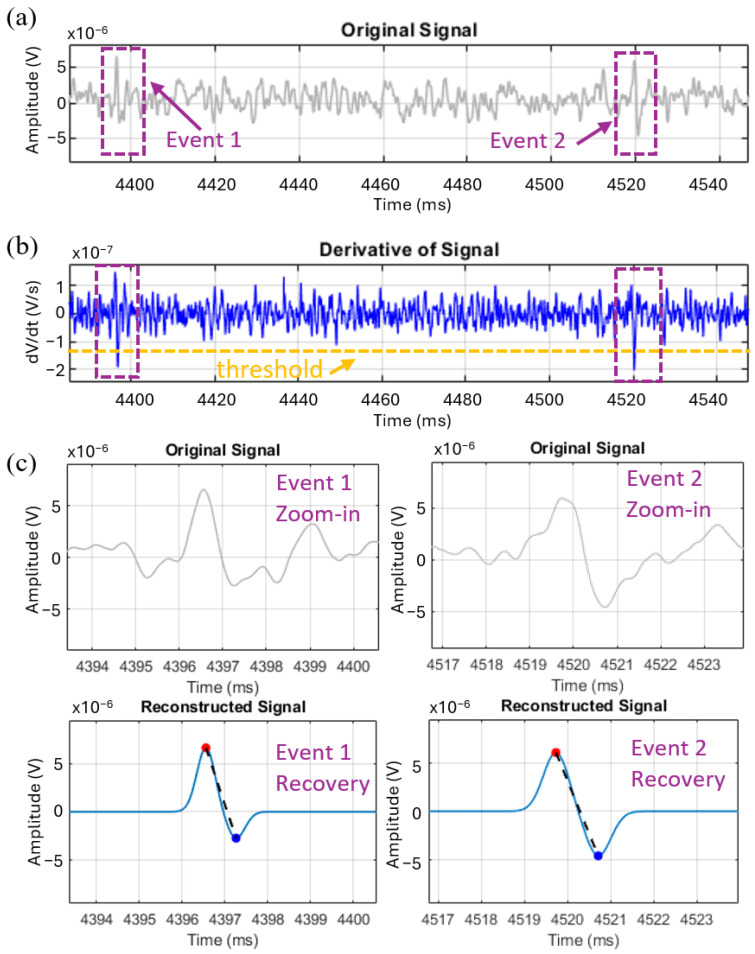
Derivative-based signal processing of impedance flow cytometry data. (**a**) A short segment of a demodulated impedance trace showing two events of interest, where the purple dashed boxes indicate the regions corresponding to particle passages; (**b**) Derivative of the signal with an orange dashed horizontal line representing the threshold used for event detection; (**c**) Zoomed-in views of the original signal for Event 1 and Event 2 (**top**), and their recovery profiles with identified peaks (**bottom**), where the solid blue curve represents the reconstructed signal, the red and blue markers denote the detected peak positions, and the black dashed line connects the peak pair for each event.

To demonstrate the effectiveness of the proposed derivative-based method for event detection and signal reconstruction under realistic noise conditions, we applied the algorithm to experimental data acquired using 2 µm polystyrene beads. As shown in Figure 2a, the original signal segment contains transient bipolar events that are partially obscured by overlapping low-frequency noise components. By computing the first derivative (Figure 2b), slope-based transitions corresponding to event boundaries become more distinguishable. A fixed threshold applied to the derivative identifies significant transients indicative of particle passage. Notably, Figure 2a highlights two events that are obscured by background fluctuations arising from low SNR and baseline drift, making direct thresholding of the raw signal challenging. In contrast, the derivative-based method amplifies local slope changes while simultaneously minimizing the influence of baseline drift, thereby enabling robust event isolation and accurate signal reconstruction even under poor signal visibility.

Following detection, each signal segment is reconstructed using a parametric model to obtain a smooth approximation of the underlying waveform. In this case, a bi-Gaussian model was chosen as a representative waveform for spherical particles and two-electrode-pair configurations. Figure 2c shows zoomed-in views of two representative events and their reconstructions. The results show that the recovered bi-Gaussian waveforms closely follow the expected shape of bead passages, with well-preserved peak amplitudes and temporal symmetry. These results confirm that derivative-based detection combined with parametric reconstruction can reliably recover event morphology under challenging noise conditions. It is worth noting, however, that the performance of the method may depend on the dominant noise characteristics of the dataset. In particular, datasets dominated by the high-frequency components of white noise can pose greater challenges for the derivative method, since differentiation inherently amplifies high-frequency components.

Theoretically, the performance of the method can be understood by modeling the demodulated time-domain signal as the sum of three components: the transient IFC events $e\left(t\right)$, a slowly varying baseline drift $b\left(t\right)$, and additive measurement noise $n\left(t\right)$:

(1) $$s\left(t\right)\;=e\left(t\right)+b\left(t\right)+n\left(t\right)$$

Taking the first derivative yields

(2) $$s^{\prime }\left(t\right)=e^{\prime }\left(t\right)+b^{\prime }\left(t\right)+n^{\prime }\left(t\right)$$

Because $b\left(t\right)$ varies slowly on the event time scale, $b^{\prime }\left(t\right)\approx 0$, so the derivative acts as a high-pass operator that suppresses drift while emphasizing the sharp ingress/egress slopes of each pulse. Thresholding ${|s}^{\prime }\left(t\right)|$ isolates the paired high-slope extrema characteristic of IFC events even when raw amplitudes are partially buried.

### 3.2. Performance Evaluation

**Figure 3:**
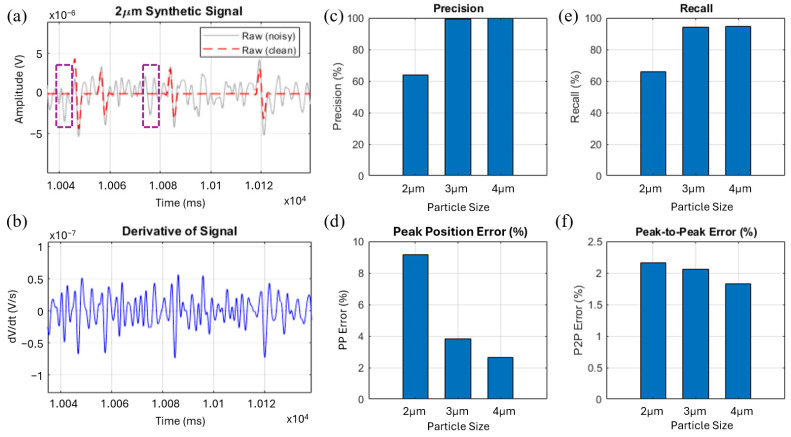
Event detection performance metrics of the derivative-based method across varying particle sizes. (**a**) Synthetic 2 µm impedance amplitude signal with noisy and clean traces indicated, where the dashed boxes indicate signal fluctuations that could be falsely identified as particle events by a simple threshold-based method; (**b**) Derivative of the signal highlighting slope-based event features; (**c**) Precision comparison for 2 µm, 3 µm, and 4 µm particles; (**d**) Peak position error (%) across particle sizes; (**e**) Recall comparison for 2 µm, 3 µm, and 4 µm particles; (**f**) Peak-to-peak error (%) across particle sizes.

The robustness and efficiency of the proposed derivative-based method were systematically evaluated across a range of synthetic datasets that closely reflected the noise characteristics of our experimental recordings. Figure 3a,b illustrate an example of the difference between the raw signal and its derivative. While the raw signal exhibits fluctuations that can be mistakenly identified as events by a simple threshold-based method, the derivative approach effectively suppresses these spurious detections. Two potential false positive events are highlighted in the raw trace, which would likely be misclassified as a particle passage under simple thresholding. After applying the derivative, these artifacts become more separable from genuine events, which makes them easier to reject during detection and significantly improves robustness in noisy environments.

As expected, performance improves with particle size due to increased signal amplitude and SNR. As shown in Figure 3c–f, both precision and recall rise markedly as particle size increases from 2 µm to 4 µm while both peak position error and peak-to-peak amplitude error decrease correspondingly. These results confirm that the derivative-based method scales consistently with particle size and behaves as anticipated across different SNR regimes.

**Figure 4:**
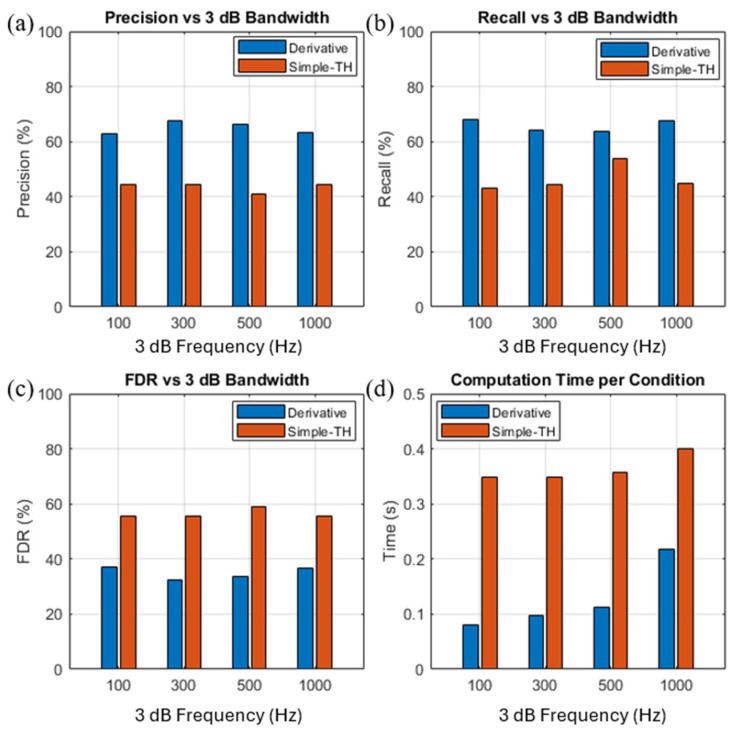
Event detection performance metrics of the derivative-based method across varying flow rate and low-pass filter settings for 2 µm particles. (**a**) Precision versus 3-dB low-pass bandwidth for the derivative and simple thresholding methods; (**b**) Recall versus 3-dB bandwidth under the same conditions; (**c**) False detection rate (FDR) versus 3-dB bandwidth; (**d**) Computation time per condition for both methods.

To rigorously compare the proposed derivative-based approach with the simple thresholding-based method, we evaluate both methods on a challenging synthetic dataset consisting of 2 µm particles processed under varying flow rates and low-pass filter bandwidths (3-dB cutoff frequencies). Flow rate was adjusted to modulate event transit time and dominant frequency content, while the low-pass bandwidth was varied to balance noise suppression and waveform fidelity; sweeping both parameters emulates realistic operating conditions and tests robustness as event durations and SNR change (see Table 2 for the bandwidth–flow-rate pairs). For a fair comparison, thresholds for both methods were tuned to approximately balance precision and recall and avoid under- or over-detection. As shown in Figure 4a–d, the derivative method consistently outperforms simple thresholding across all cutoff frequencies, improving precision and recall by ~20% and reducing the false discovery rate (FDR) by 15–25%, while requiring only 22–55% of the computation time. These results show that the derivative method effectively suppresses false positives, preserves sensitivity, and significantly reduces computation time. Its stable performance across cutoff frequencies and flow rate further demonstrates its adaptability to diverse acquisition conditions.

While the derivative method is robust across practical bandwidth conditions, the results also reveal its inherent limitations. At low cutoff frequencies (e.g., 100 Hz), the narrow bandwidth combines with the slow flow-rate condition to produce long, heavily smoothed event waveforms with flattened slopes. As discussed in Section 3.1, the derivative operator suppresses slow baseline drift but is also sensitive to the sharpness of local transitions; therefore, this slope attenuation reduces boundary contrast and makes the derivative more vulnerable to small residual fluctuations. These effects are reflected in the reduced precision and elevated FDR observed at 100 Hz (Figure 4a,c). At the opposite extreme, when the 3-dB cutoff is very wide (e.g., 1000 Hz), more high-frequency noise passes through the front-end filter, and differentiation amplifies these components. This increases the number of noise-induced excursions above the detection threshold, again leading to higher FDR and slightly reduced precision relative to mid-range bandwidths (Figure 4a,c). Interestingly, despite the poorer precision and higher FDR at these bandwidth extremes, recall is relatively high at both 100 Hz and 1000 Hz. We hypothesize that in the 100 Hz case, the slow, broadened events produce extended derivative excursions, which can trigger multiple threshold crossings for the same true event, reducing false negatives but increasing false positives. In the 1000 Hz case, high-frequency noise generates additional crossings that overlap with true events, again lowering false negatives while simultaneously inflating false positives. Thus, the elevated recall at 100 Hz and 1000 Hz reflects over-detection rather than genuinely improved sensitivity, consistent with the corresponding rise in FDR. Although the derivative method still outperforms simple thresholding under all tested conditions, these observations indicate that extremely narrow or extremely wide bandwidth settings may require a small smoothing window or hardware-level filtering to maintain stable derivative signals in real-time applications. In addition, the derivative operation must be evaluated on every sample in the raw trace, meaning the computational load scales linearly with sampling rate. In our tests, this cost remained minimal: the derivative pipeline required only 22–55% of the computation time of the multi-stage thresholding method, completing in ~0.08–0.22 s for a 30 s data record. However, at substantially higher sampling rates—such as multi-MHz acquisition systems—this per-sample processing requirement may place additional load on resource-limited microcontrollers. In such scenarios, down sampling, lightweight smoothing, or hardware-assisted differentiation can mitigate the computational impact.

### 3.3. Real-Time Classification Performance

To evaluate the feasibility of deploying the proposed derivative-based method for real-time particle classification, we integrated it into a complete signal processing and machine learning pipeline, evaluated first on synthetic IFC data. Synthetic data streams were generated to emulate realistic experimental conditions, containing randomly distributed events for 4 μm and 7 μm beads. Event detection and feature extraction—specifically peak-to-peak magnitude and transit time—were performed entirely by the derivative-based algorithm and used as inputs for classification. For model training, 30 synthetic data streams were used, encompassing multiple bead classes. The full training workflow—including synthetic data generation, derivative-based detection, and feature extraction—was completed within the order of seconds, while the model-fitting step itself required only a negligible fraction of this time. This efficiency arises from the decision-tree classifier operating on low-dimensional, highly discriminative derivative-based features. The same computational advantages extend to the inference stage. Because both event detection and feature extraction modules operate in strict linear time (O(N)) with minimal memory overhead, the processing time using the derivative-based framework is roughly two orders of magnitude faster than the rate of data acquisition. This ensures ample headroom for real-time or resource-constrained IFC implementations.

**Figure 5:**
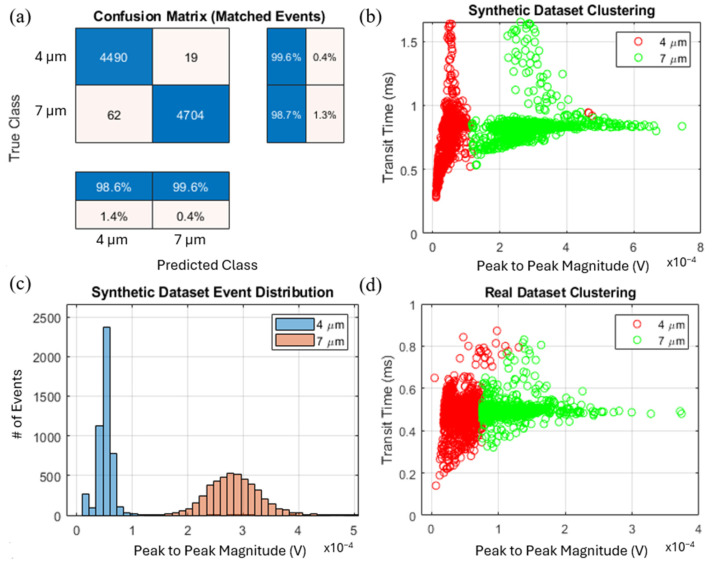
Real-time machine learning-assisted classification of 4 μm and 7 μm beads using synthetic and experimental datasets. (**a**) Confusion matrix summarizing classification accuracy; (**b**) Clustering of synthetic dataset events based on extracted features (transit time and peak-to-peak magnitude). (**c**) Event distribution for the synthetic dataset; (**d**) Clustering of real experimental events.

Classification performance is summarized in Figure 5a, where the confusion matrix shows that precision and recall exceeded 98.6% for both bead sizes. The clustering of features (Figure 5b) and corresponding event distributions (Figure 5c) further demonstrate the strong discriminative power of the extracted features, with minimal class overlap.

To assess its generalizability under real-world conditions, we applied the same pipeline to experimental data collected from a mixed suspension of 4 μm and 7 μm polystyrene beads. Signals were acquired using a microfluidic device with a differential electrode pair and processed with the derivative-based detection algorithm. The classifier—trained exclusively on synthetic data—was applied directly to the experimental dataset without retraining. As shown in Figure 5d, the model successfully classified the events into two distinct populations corresponding to 4 μm and 7 μm beads, demonstrating strong zero-shot transfer from simulation to experiment. Minor shifts in the experimental feature distribution likely arise from inherent size variance in commercial bead samples, slight concentration inhomogeneities during sample preparation, and small flow-rate fluctuations in the physical measurements.

## 4. Conclusions

In this study, we present an integrated derivative-based framework for IFC that combines baseline-drift removal, event detection, and feature extraction into a single, computationally lightweight operation. By applying the first derivative to the demodulated time-domain signal, the proposed method effectively enhances transient slope features of IFC events while attenuating baseline drift. This design directly targets key limitations of threshold-based schemes and offers a more robust alternative under challenging signal-to-noise conditions.

Systematic evaluations across a wide range of flow rates (0.3–3 µL min^−1^) and low-pass filter bandwidths (100–1000 Hz) demonstrate that the derivative-based method consistently outperforms the simple amplitude thresholding method. For 2 µm beads, the proposed framework improves precision and recall by approximately 20% and reduces the FDR by 15–25%, while requiring only 22–55% of the processing time across all tested conditions. The algorithm also exhibits outstanding computational efficiency and strong compatibility with real-time machine learning workflows. When paired with a pretrained lightweight classifier, the complete derivative-based pipeline operates roughly two orders of magnitude faster than the rate of data acquisition, while achieving > 98.6% classification precision and recall in distinguishing 4 µm and 7 µm synthetic particles. These results confirm both the strong discriminative power of the extracted features and the exceptionally low computational overhead.

Experimental validation using a mixed suspension of 4 µm and 7 µm polystyrene beads further confirms the method’s generalizability. The same model trained exclusively on synthetic data successfully classified experimental events without retraining, demonstrating strong zero-shot transfer and reliable feature separation in real measurements. Overall, these findings demonstrate the derivative-based frameworks as a fast, accurate, and computationally lightweight solution for a real-time IFC analysis framework. Those features make it well-suited for future on-chip implementations in portable or low-power microfluidic cytometry systems.

## Figures and Tables

**Table 1:** Parameter values used in the generation of the synthetic data stream.

D (µm)	CV	ρ (#/µL)	∅ (µL/min)	n	BW (Hz)	$f_{s}$ (kSa/s)	$\sigma_{N}$ (μV)
2, 3, 4, 7	5%	$1\times 10^{3}$	2	4	500	115.1	1.3

**Table 2:** Low-Pass Filter Bandwidth Settings by Flow Rate for Synthetic Data Generation.

∅ (µL/min)	BW (Hz)
0.3	100
1	300
2	500
3	1000

## Data Availability

The raw data supporting the conclusions of this article will be made available by the authors on request.
